# Incidence, severity, and temporal development of oral complications in pediatric allogeneic hematopoietic stem cell transplant patients – a multicenter study

**DOI:** 10.1007/s00520-023-08151-1

**Published:** 2023-11-16

**Authors:** Monica Barr Agholme, Göran Dahllöf, Johan Karlsson Törlén, Alessandra Majorana, Michael T. Brennan, Inger von Bültzingslöwen, Poh Lin Tan, Shijia Hu, Yu Fan Sim, Catherine Hong

**Affiliations:** 1 Division of Orthodontics and Pediatric Dentistry, Department of Dental Medicine, Karolinska Institutet, Huddinge, Sweden; 2 Center for Oral Health Services and Research, Mid-Norway (TkMidt), Trondheim, Norway; 3 Cellular Therapy and Allogeneic Stem Cell Transplantation (CAST), Karolinska Comprehensive Cancer Center, Karolinska University Hospital, Huddinge, Sweden; 4 Department of Clinical Science, Intervention and Technology (CLINTEC), Karolinska Institute, Stockholm, Sweden; 5 Department of Pediatric Dentistry, School of Dentistry, University of Brescia, Brescia, Italy; 6 Department of Oral Medicine/Oral & Maxillofacial Surgery, Atrium Health Carolinas Medical Center, Charlotte, NC USA; 7 Department of Otolaryngology/Head and Neck Surgery, Wake Forest University School of Medicine, Winston Salem, NC USA; 8 Department of Oral Microbiology and Immunology, Institute of Odontology, The Sahlgrenska Academy, University of Gothenburg, Gothenburg, Sweden; 9 Department of Paediatrics, Yong Loo Lin School of Medicine, National University of Singapore, Singapore, Singapore; 10 Khoo Teck Puat-National University Children’s Medical Institute, National University Hospital, National University Health System, Singapore, Singapore; 11 Discipline of Orthodontics and Paediatric Dentistry, Faculty of Dentistry, National University of Singapore, Singapore, Singapore

**Keywords:** Cancer, Children, Dysphagia, Hematological malignancies, Nausea, Neutropenia, Oral mucositis, Pain, Patient-reported outcomes, Prospective

## Abstract

**Purpose:**

Oral mucositis is a common complication for patients undergoing allogeneic hematopoietic stem cell transplantation (HSCT) and causes pain and difficulties in functions like eating and swallowing, resulting in lower quality of life and greater need of treatment with opioids and parenteral nutrition. This prospective multicenter study focused on pediatric recipients of HSCT in the neutropenic phase concerning oral complications, timing, severity, and patient experience.

**Methods:**

The cohort comprised 68 patients, median age 11.1 years (IQR 6.3) receiving allogeneic HSCT at three clinical sites. Medical records were retrieved for therapy regimens, concomitant medications, oral and dental history, and subjective oral complaints. Calibrated dentists conducted an oral and dental investigation before HSCT. After HSCT graft infusion, study personnel made bedside assessments and patients filled out a questionnaire once or twice a week until neutrophil engraftment.

**Results:**

We followed 63 patients through the neutropenic phase until engraftment. 50% developed oral mucositis of grades 2–4. Peak severity occurred at 8–11 days after stem cell infusion. Altogether, 87% had subjective oral complaints. The temporal distribution of adverse events is similar to the development of oral mucositis. The most bothersome symptoms were blisters and oral ulcerations, including mucositis; 40% reported severe pain and major impact on activities of daily living despite continuous use of opioids.

**Conclusion:**

This study highlights the burden of oral complications and their negative effect on the health and quality of life of HSCT recipients.

**Supplementary Information:**

The online version contains supplementary material available at 10.1007/s00520-023-08151-1.

## Introduction

Hematopoietic stem cell transplantation (HSCT) is a curative treatment option for children and adolescents with hematologic cancer or a variety of non-malignant diseases [1–3]. With improvements in treatment modalities and supportive care, the long-term survival rate of pediatric HSCT continues to grow [4].

Pre-transplant treatment exposure, HSCT conditioning regimens, and other transplant-related events cause a range of acute and late adverse effects [5, 6]. Conditioning regimens are tailored to the diagnosis, age, and general medical status of the patient. The general aim of conditioning is to eradicate abnormal cells and to suppress the immune system in the host in order to prevent immunological graft rejection. The regimens are cytotoxic and induce a transitional phase of neutropenia, which can last for 2–4 weeks after infusion of the donated cells. During the aplastic period, the patient has low functioning immune function. The toxicity of the conditioning regimen disrupts normal mucosal and skin barriers, allowing invasion of endogenous bacteria [7, 8].

During this period, the oral cavity is vulnerable to adverse effects such as oral mucositis (OM), hyposalivation, xerostomia, taste changes, and oral infections. These occur frequently, often with sequelae that include oral pain; hypersensitivity of the oral mucosa; and difficulties in eating, drinking, and swallowing. Nutritional status and oral medication compliance can be negatively affected [9–11].

Previous studies on oral complications during the neutropenic phase in children and adolescents are few, often retrospective, and include small patient samples. One of these is the study of Doss et al. [12], who found, in their examination of 19 pediatric HSCT recipients, that gingivitis, plaque accumulation, mucositis, and oral ulcerations were common after pediatric HSCT.

Due to the lack of prospective studies on oral complications during the neutropenic phase in children and adolescents, particularly regarding the timing and severity of OM and patient-reported outcome measures, we conducted this multicenter prospective study on oral complications in pediatric HSCT recipients.

## Patients and methods

### Patients

This study is a prospective longitudinal multicenter cohort study involving children and adolescents undergoing allogeneic HSCT at three centers: Karolinska University Hospital (Huddinge, Sweden), Atrium Health Carolinas Medical Center (Charlotte, North Carolina, USA), and the National University Hospital (Singapore). Three authors (CH, IvB, MTB) oversaw all centers to ensure an infrastructure, patient population, and research staffing that complied with the study design as well as calibration of research personnel in all procedures, including data management and study outcomes [13].

Children and adolescents who were routinely referred for a dental evaluation before HSCT were invited to participate in the study between May 2015 and December 2019. Supplementary Fig. 1 illustrates a flow-chart of patients eligible for the study and their reasons for dropping out. Patients able to cooperate during dental examinations, answer questions and interpret meaning of pictures shown to them were included; their parents or guardians signed informed-consent forms after this initial evaluation. Therefore no exact age limit was set, the youngest included child was 4.5 years. Data on children admitted to the transplant centers but not referred for a dental examination was not collected. Regarding the 18 patients excluded (Supplementary Fig. 1), data is available for 14. The mean age was 3.5 ± 3.7 years (range 2–9), diagnoses were ALL/AML 7, myelodysplastic syndromes 2, hematological non-malignant 4 and other non-malignant 1.

### Donor matching, stem cell source, and cell dose

This study included only allogeneic HSCT recipients. Among donors, 35% were HLA-matched related (i.e., allelic matches in ≥ 8 HLA Class I and II loci), 24% were HLA-matched unrelated, 35% were HLA-mismatched related, and 6% were mismatched unrelated. Stem cell grafts were sourced from peripheral blood, bone marrow, or cord blood. Patients were grouped according to dose: ≤ 10 × 10^6^ or > 10 × 10^6^ CD34 + cells/kg. Most patients (60%) received bone marrow cells after conditioning while 35% received stem cells derived from peripheral blood (Table 1).

**Table 1:** Characteristics of the child and adolescent patients (n = 68) scheduled for allogeneic hematopoietic stem cell transplantation and their transplant characteristics

Variables	n	%
Age, years (median, IQR)	11.1, 6.3	
Sex (female/male)	22/46	32/68
Underlying disease		
Acute lymphoblastic leukemia	21	31
Acute myeloid leukemia	14	20
Myelodysplastic/Myeloproliferative syndromes	6	9
Lymphoma	3	4
Hematological non-malignant	23	34
Other non-malignant	1	2
Transplant donor		
HLA-matched sibling	24	35
HLA-matched unrelated	16	24
HLA-mismatched related	24	35
HLA-mismatched unrelated	4	6
Stem cell source		
Peripheral blood	24	35
Bone marrow	41	60
Cord blood	3	4
Conditioning regimen^a^		
Reduced conditioning	12	18
Myeloablative conditioning	53	82
Graft-versus-host disease prophylaxis^b^		
Calcineurin inhibitors alone	11	17
Calcineurin inhibitors combined with other drugs	52	83

IQR: Interquartile range; HLA: human leukocyte antigen; ^a^data missing for three patients; ^b^data missing for five patients

### Conditioning

Patients included in this study underwent several protocols [14]. Based on current standards, our conditioning protocols were of two types: myeloablative conditioning (MAC) and reduced intensity aconditioning (RIC). The MAC protocols usually included total body irradiation while the RIC protocols included fludarabine and intermediate doses of alkylating agents such as melphalan and busulfan [15–17]. Of the study patients, 56% received MAC and 44% followed a RIC protocol [14].

### Immunosuppressive prophylaxis

Graft-versus-host disease (GVHD) prophylaxis regimens were of two standard types: those including only calcineurin inhibitors, and those including calcineurin inhibitors in combination with other drugs [18]. Depending on patient and donor characteristics, some of the patients in these groups also received anti-thymocyte globulin or other T-cell depletion protocols as part of their conditioning [19].

### Viral serology and prophylaxis

Before HSCT, all recipients and donors were screened for herpes virus seropositivity: cytomegalovirus (CMV), herpes simplex viruses (HSV), and varicella zoster virus (VZV).

### Acute graft-versus-host disease

Acute GVHD was assessed clinically and assigned a grade from 0 to 4 according to criteria published in 1995 by Przepiorka et al. [20].

### Oral examinations

The baseline dental examination of the oral cavity included a medical and dental history, medications, and patient reports of oral problems. About 14 days before HSCT, patients were examined clinically. The exam included inspections of the oral mucosa, teeth, and saliva; subjective oral complaints were noted and objective measurements were made. Treatment was performed when necessary. All children and adolescents and their parents were thoroughly instructed in the importance of oral self-care pre- and post-transplant. The standard oral hygiene protocol included careful tooth brushing twice a day with a very soft toothbrush. Patients were advised to suck on ice chips throughout conditioning, if possible, and to lubricate their lips as protection against dehydration and cracking. When experiencing OM, patients were advised to rinse their mouth with saline [17].

### Oral bedside examination and oral mucositis assessments

After graft infusion, a bedside assessment was done once or twice a week for at least 14 days post-HSCT, and longer if an absolute neutrophil count > 0.5 × 10^9^ had not been reached. To assess subjective oral complications and functions, we queried patients about any oral pain, dry mouth symptoms, changes in taste, swallowing difficulties, nausea, and poor nutrition and about parenteral nutrition. A dentist made an objective examination of the oral mucosa following the World Health Organization (WHO) grading scale for OM [21]. WHO scoring includes subjective and functional outcomes (pain and ability to eat) and objective signs of mucositis (ulceration and erythema); possible scores were 0 – no findings; 1 – erythema and soreness, no ulcers; 2 – oral erythema, oral ulcers, solid diet tolerated; 3 – oral ulcers, liquid diet only; and 4 – oral ulcers, unable to tolerate a solid or liquid diet.

### Subjective complaints

The study protocol included a guide for the bedside interviews. Before study start, all examiners were calibrated in use of the WHO mucositis scale. Examiners were also calibrated in the administration of the Children’s International Mucositis Evaluation Scale. Besides the items on the WHO scale, our assessment included other questions on subjective complaints [22, 23] (Supplementary Table 1).

### Data management

Patient data were entered into MedView, a software program designed for clinical research and suited for multicenter studies [24].

### Ethical considerations

When invited to participate, all patients and their parents received oral and written information about the study. The children received a simplified version of the information, with pictorial support. Adolescents were involved in the discussion about their study participation. Before any assessments were done, the parents and the adolescents signed an informed-consent form concerning the research and publication of results.

The local ethics committees at each site approved the study protocol: Stockholm, Sweden, daybook no. [DNR] 2016/757; Charlotte, NC, USA, institutional review board [IRB] daybook no. 000884668; and Singapore, National University of Singapore [NUS] daybook no. 2012/00229. The study conformed to the ethical principles of the Declaration of Helsinki. All authors attest the accuracy of the reported data.

### Statistical analyses

Descriptive statistics (frequency and percentage for qualitative data; median and interquartile range [IQR] for quantitative data) were used to summarize information collected. Comparisons of the prevalence of mucositis and transplant characteristics were done with Fisher’s exact test. A comparison of the age distribution of those with and without OM (WHO grades 0–1 vs grades 2–4) was done with the Mann–Whitney U test. Mixed effect logistic regression model was performed to determine factors associated with OM. A backward elimination approach with removal threshold set at a *p*-value > 0.1 was used. Statistical significance was set at *p* < 0.05, and all *p*-values were two-sided. All statistical analyses were done using R 4.2.2 (R core Team, 2022) and lme4 (v1.1.31) package [25].

## Results

During 2015–2019, 68 children and adolescents were recruited to the study (Stockholm [*n* = 28], Charlotte [*n* = 10] and Singapore [*n* = 30]). Before receiving HSCT, 95 patients were referred for evaluation; 71 met the inclusion and exclusion criteria and agreed to participate. The excluded patients were younger with a similar distribution of diagnoses. Before conditioning, however, 2 patients withdrew, and 1 died (Supplementary Fig. 1).

Of the remaining 68 (median age 11.1 years [range 4.5–19.8; IQR 6.3]; male: *n* = 46 [68%]), we followed 63 (93%) throughout the neutropenic period until the neutrophils had recovered to > 0.5 × 10^9^. The most common diagnoses were acute lymphoblastic leukemia (ALL, 32%), acute myeloid leukemia (AML, 21%), and other non-malignant hematological diseases (41%; Table 1).

**Fig. 1:**
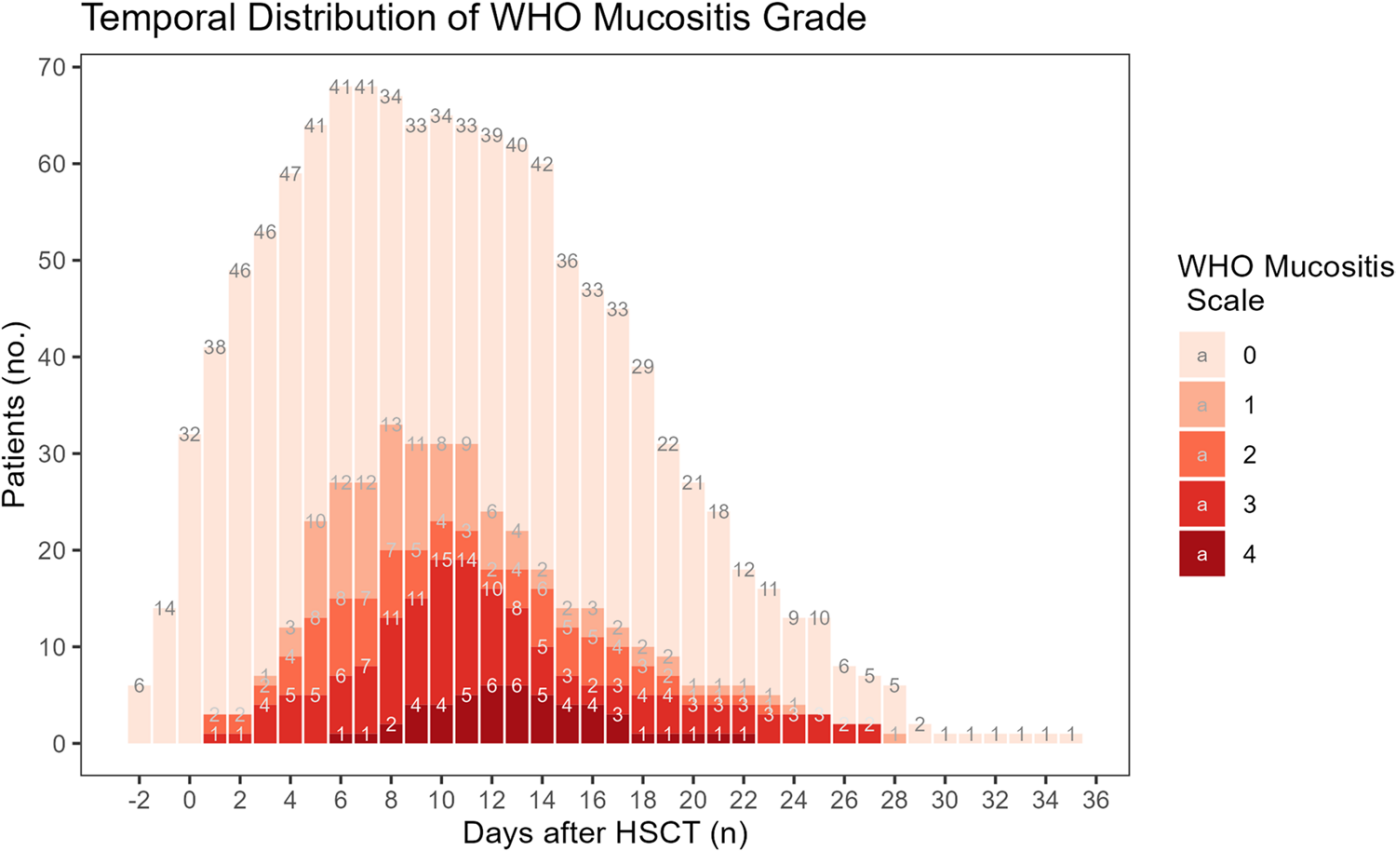
Temporal development of mucositis severity following hematopoietic stem cell transplantation (HSCT). Grade 0: no findings; 1: erythema and soreness, no ulcers; 2: oral erythema, oral ulcers, solid diet tolerated; 3: oral ulcers, liquid diet only; 4: Oral ulcers, unable to tolerate a solid or liquid diet

During the neutropenic phase, 34 (50%) patients developed OM grades 2–4. Figure 1 presents the temporal development (number and severity). The first signs of OM were observed the first day after receiving HSCT. The peak incidence of OM occurred on days + 8–11 and peak severity occurred on day + 10. On this day, distribution of OM was 6% (grade 4); 23% (grade 3); 6% (grade 2); and 12% (grade 1); slightly more than half (53%) did not have OM. It also seems that the more severe forms peaked later, at 12–13 days post-transplant. OM development was steep up to peak incidence; post-peak, resolution (healing) was slower in patients with OM grades 2–4. No ulcerative OM was diagnosed after day + 27. The lowest white blood cell count occurred on day + 8 post-transplant.

Table 2 presents a univariate analysis of background and treatment-related factors comparing patients with the severe OM grades of 2–4 with the less severe OM grades 0–1. Patients diagnosed with OM grades 2–4 were significantly older compared to those with OM grades 0–1 (*p* = 0.042). No differences in OM severity were observed between those with matched donors compared to those with mismatched donors or those receiving MAC and those receiving RIC. Neither did we find any significant differences in OM severity concerning recipient or donor seropositivity of CMV, HSV, VZV, or combinations thereof (data not shown).

**Table 2:** Patient and transplant characteristics in allogenic hematopoietic stem cell transplants and the child and adolescent recipients (*n* = 68). The WHO grading scale for oral mucositis was used to group patients according to severity: grades 0–1 (*n* = 34) and grades 2–4 (*n* = 34)

Characteristics	OM severity group (%)		*p*-value
	Grades 0–1	Grades 2–4	
Age (years: median, Q1–Q3)	10.2 (7.5–12.5)	12.8 (9.8–15.1)	**0.042**^d^
Sex (male/female)			
Male	20 (44%)	26 (56%)	0.194^e^
Female	14 (64%)	8 (36%)	
Underlying disease			
Hematologic, malignant	20 (44%)	26 (56%)	0.229^e^
Hematologic, non-malignant	10 (59%)	7 (41%)	
Other	4 (80%)	1 (20%)	
Transplant donor			
HLA-matched	16 (40%)	24 (60%)	0.084^e^
HLA-mismatched	18 (64%)	10 (36%)	
Stem cell source^a^			
Peripheral blood	15 (65%)	8 (35%)	0.155^e^
Bone marrow	17 (42%)	24 (58%)	
Cord blood	1 (50%)	1 (50%)	
Stem cell dose (× 10^6^/kg)^b^			
≤ 10	12 (40%)	18 (60%)	0.273^e^
> 10	14 (58%)	10 (42%)	
Conditioning regimen^c^			
Reduced intensity	5 (42%)	7 (58%)	0.750^e^
Myeloablative	27 (52%)	25 (48%)	
Graft-versus-host disease prophylaxis^d^			
Calcineurin inhibitors alone	5 (46%)	6 (54%)	1.000^e^
Calcineurin inhibitors in combination with other drugs	25 (48%)	27 (52%)	

OM: oral mucositis; HLA: human leukocyte antigen; ^a^data missing for 2 patients; ^b^data missing for 14 patients; ^c^Data missing for 4 patients; ^d^data missing for 5 patients; ^d^Mann–Whitney U Test; ^e^ Exact Test; significant values in bold

A multivariate analysis found malignant disorders (OR = 0.22; 95% CI 0.06–0.91; *p* = 0.037) and matched donors (OR = 0.21; 95% CI 0.06–0.71: *p* = 0.013) to be significantly associated with OM grades 2–4 (Supplementary Table 2).

### Subjective complaints

Table 3 presents subjective complaints related to OM severity. During the neutropenic phase, 59 (87%) had subjective complaints from the mouth and 19 (8%), symptoms involving the teeth. Patients with OM grades 2–4 generally had more subjective oral complaints compared to those with minor or no OM. When asked how uncomfortable the worst symptom was, 26 (76%) of those with OM grades 2–4 reported ≥ 3 on a Wong Baker faces scale [21] compared to 10 (29%) of those with OM grades 0–1 (*p* < 0.001).

**Table 3:** Self-reported subjective symptoms during the neutropenic phase in allogeneic hematopoietic stem cell transplant patients (*n* = 68)

Symptoms	Oral Mucositis Grade 0/1^a^ (*N* = 34)	Oral Mucositis Grade 2/3/4 (*N* = 34)	*p*-value^c^
Do you have any symptom(s) that bothers you in the oral cavity?			**0.027**
No	8 (24%)	1 (3%)	
Yes	26 (76%)	33 (97%)	
Symptoms involve the teeth			1.000
No	25 (74%)	24 (71%)	
Yes	9 (26%)	10 (29%)	
Symptoms involve throat sensitivity			**0.003**
No	20 (59%)	7 (21%)	
Yes	14 (41%)	27 (79%)	
Symptoms involve taste changes			0.087
No	19 (56%)	11 (32%)	
Yes	15 (44%)	23 (68%)	
Worst/most bothersome oral symptoms			0.512
Dry mouth			
No	30 (88%)	27 (79%)	
Yes	4 (12%)	7 (21%)	
Blister/Ulceration/Mucositis			** < 0.001**
No	33 (97%)	19 (56%)	
Yes	1 (3%)	15 (44%)	
Taste Changes			1.000
No	23 (68%)	22 (65%)	
Yes	11 (32%)	12 (45%)	
Throat sensitivity			**0.028**
No	23 (68%)	13 (38%)	
Yes	11 (32%)	21 (62%)	
How uncomfortable/painful is your worst oral symptom?^b^			** < 0.001**
Grades 0–2	24 (71%)	8 (24%)	
Grades 3–5	10 (29%)	26 (76%)	
How does oral pain impact your daily functioning?^b^			** < 0.001**
Grades 0–2	29 (85%)	12 (35%)	
Grades 3–4	5 (15%)	22 (65%)	
Current analgesic use^b^			**0.004**
Grades 0–2	17 (50%)	5 (15%)	
Grades 3–4	17 (50%)	29 (85%)	
Dry mouth^b^			**0.045**
Grades 0–1	32 (94%)	25 (74%)	
Grades 2–3	2 (8%)	9 (26%)	
What can the patient eat?			0.614
Solid food	33 (97%)	31 (91%)	
Liquid food	1 (3%)	3 (9%)	
Nothing by mouth	0 (0%)	0 (0%)	

^a^The WHO grading scale for oral mucositis [20];^b^ self-report questionnaire: Supplementary Table 1. ^**c**^ Exact test, significant values in bold

Use of analgesics was frequent, and 29 (85%) of those with OM grades 2–4 required continuous use of opioids compared to 17 (50%) with OM grades 0–1 (*p* < 0.014). Twenty-two (65%) children diagnosed with OM grade > 1 reported severe pain and a major impact on their activities of daily living (ADL) compared to 5 (15%) of those with OM grades 0–1 (*p* < 0.001).

**Fig. 2:**
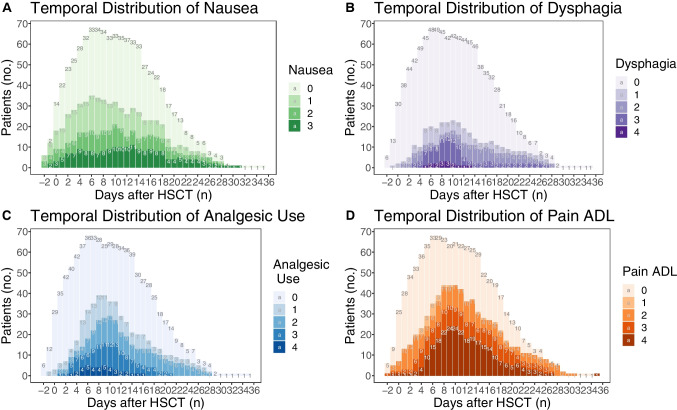
**A**. Temporal development of nausea following hematopoietic stem cell transplantation (HSCT): (0) no problem, (1) loss of appetite without changing eating habits, (2) decreased food intake, (3) loss of appetite with reduced drink and food intake; also, tube feeding or total parenteral nutrition. **B.** Temporal development of dysphagia following hematopoietic stem cell transplantation (HSCT): (0) no problem, (1) symptoms, but can eat as usual, (2) more symptoms, can drink but not eat, (3) insufficient nutrient supply, cannot drink or eat, tube feeding or total parenteral nutrition, (4) potentially life threatening. **C.** Temporal development of analgesic use following hematopoietic stem cell transplantation (HSCT): (0) none, (1) topical anesthesia, (2) peroral non-steroidal anti-inflammatory drugs, (3) peroral opioids, (4) intravenous opioids. **D**. Temporal development of oral pain and impact on daily functioning following hematopoietic stem cell transplantation (HSCT): (0) no problem, (1) mild pain, does not impact functioning, (2) moderate pain, affects functioning but not activities of daily living (ADL), (3) severe pain that affects ADL, (4) potentially life-threatening pain [22]

The temporal distributions of nausea (Fig. 2A), dysphagia (Fig. 2B), use of analgesics (Fig. 2C), and pain impact on daily functioning (Fig. 2D) have similar distributions of severity, likewise the OM index. Dysphagia, use of analgesics, and impact on ADL peaked at + 10 days post-transplant, had a steeper increase to peak, and showed a slower resolution of symptoms. Nausea, on the other hand, developed even faster and peaked at day + 6 post-transplant; the duration of more severe effects was also longer (Fig. 2A). Some patients suffering from dysphagia still had difficulties eating solid foods at 28 days post-transplant (Fig. 2B). Use of analgesics peaked at day + 10, mirroring the peak incidence of OM grades 2–4. Three weeks post-transplant, all patients had discontinued all types of opioids (Fig. 2C). Figure 2D shows the extent to which the patients report severe limitations in their ADL due to oral pain and discomfort.

## Discussion

The results of this prospective multi-center study of oral complications during the neutropenic phase of pediatric HSCT showed that half of the children developed OM grades 2–4 post-transplant. Peak severity occurred on days + 8–11 after stem cell infusion. Altogether, 87% reported subjective oral symptoms. The distribution of adverse events had a temporal distribution similar to the development of OM. The most bothersome symptoms were blisters, oral ulcerations including mucositis, and severe pain that had a major impact on ADL despite ongoing use of opioids.

This study is part of an international study of oral complications in allogeneic HSCT recipients. The protocols were developed in a group effort based on previous research. Most patients had been diagnosed with hematological disorders, both malignant and non-malignant, which are the most common indication for HSCT in children and adolescents [3]. In this study, we defined OM grades 2–4 as severe OM; WHO defines grades 3–4 as severe OM. The rationale behind our classification of grade 2 as severe OM is that ulcerations occur more frequently in children and adolescents than in adults, which is attributed to the higher rate of cell division in their oral mucosa [26], and that ulcers constitute a port of entry for early infections [27].

During the neutropenic phase, half the patients developed OM grades 2–4. The Doss et al. [12] study on 19 patients reported that 68% developed mucositis and 58% had oral ulcerations during the first 28 days post-transplant. In a retrospective study of 45 children, of which 24 were treated with allogeneic HSCT, 71% developed severe mucositis, which is higher than in our study [11].

Peak incidence of severe OM in the neutropenic phase for HSCT recipients varies in the literature. Doss et al. [12] reported a peak on day + 7 in children and adolescents; however, they examined the oral cavity once every seven days. Garming Legert et al. [17] reported a peak incidence on days + 10–11 in adults. Our study also observed a peak incidence of severe OM on days + 8–11 post-transplant.

The important contribution of our study is an illustration of the temporal development of OM throughout the neutropenic period. On days + 8–11 post-transplant, 47% of the patients were diagnosed with OM of a grade ˃ 0, of which 6% were diagnosed with the most severe form, grade 4, including extensive ulcerations that made oral intake of drinks and food impossible.

Few studies have published patient-reported outcome measures in the context of pediatric HSCT [27, 28]. The most common oral problems in the neutropenic phase were pain and sensitivity followed by swallowing problems with an inability to eat, drink, and talk. These problems often require total parenteral nutrition and use of opioid analgesics. They also increase the risk of systemic infections due to the disruption of the oral mucosal barrier, unscheduled and prolonged hospital stays, and weeks-long impacts on the quality of life. The results show that children and adolescents experience severe pain during the neutropenic period, despite use of analgesics recommended for severe cancer-induced pain [29, 30].

Pain experienced during pediatric HSCT is reported to be especially severe and complex due to the high intensity of conditioning regimens [26, 31]. Children and adolescents experience multiple painful complications as an outcome of HSCT therapy over the lengthy trajectory of their hospitalization [29]. At the same time, children and adolescents commonly hide their pain from their parents and healthcare providers. When they do communicate pain, they are motivated by an urgent need for pain intervention [32, 33].

In our study, all patients were advised to follow the oral care program suggested by the Multinational Association of Supportive Care in Cancer/International Society of Oral Oncology [34]. All patients had oral care prior to HSCT, and the caregivers and patients were encouraged to maintain optimal oral care in order to minimize oral problems and discomfort before, during, and after HSCT. Development of dental plaque and gingivitis could increase post-transplant time with more severe OM; this is an important issue in supportive care during the neutropenic phase [12]. Findings that supportive oral care in adult patients receiving HSCT decreased the occurrence and severity of OM support the maintenance of optimal oral care [35, 36].

The strengths of this study is the large cohort of participants which was achieved with the multi-center design, the prospective design and close and frequent examinations of the patients during the neutropenic phase increased the awareness of oral health. The limitations are that inter- and intra-reliability variation between examiners and centers was not measured because of the ethical problems with subjection severely ill patients to these measurements. Furthermore, a number of transplanted children with an initial dental examination were not included due to low age or inability to communicate in the native language. This may introduce a bias since the youngest patients are not included in the study.

In conclusion, this study found that 50% developed oral mucositis of grades 2–4 during the neutropenic period. Peak severity occurred at 8–11 days after stem cell infusion. Altogether, 87% had subjective oral complaints. The temporal distribution of adverse such as nausea, dysphagia and pain were similar to the development of oral mucositis.

### Supplementary Information

Below is the link to the electronic supplementary material.

Supplementary file1 (DOCX 33 KB)

## Data Availability

The datasets analyzed in this study are available from the corresponding author on reasonable request.
