# Supplementary material for: Incidence, severity, and temporal development of oral complications in pediatric allogeneic hematopoietic stem cell transplant patients – a multicenter study
Source: Support Care Cancer. 2023 Nov 16;31(12):702. doi: 10.1007/s00520-023-08151-1 (PMC10654176; doi:10.1007/s00520-023-08151-1)
Supplement: Supplementary file 1 — Supplementary file1 (DOCX 33 KB) [file 520_2023_8151_MOESM1_ESM.docx]

**Online Resource**

**Supplementary Table 1.** Questionnaire on subjective complaints during the neutropenic phase [22,23].

___________________________________________________________________________

How uncomfortable/painful is your worst/most bothersome oral symptom now

0 no problem

1 little discomfort/pain,

2 little more discomfort/pain

3 even more discomfort/pain

4 a lot of discomfort/pain

5 can’t eat/swallow/talk/sleep

Do you have oral pain, and if so how much does it impact your daily functioning?

0 no problem

1 mild pain, does not impact functioning

2 moderate pain, affects functioning but not activities of daily living (ADL)

3 severe pain that affects ADL

4 life threatening

Swallowing difficulties (dysphagia)

0 no problem

1 symptoms but can eat as usual

2 more symptoms, can drink but not eat

3 insufficient nutrient supply, cannot drink or eat, tube feeding or total parenteral nutrition

4 life threatening

Nausea

0 no problem

1 loss of appetite without changing eating habits

2 decreased food intake

3 loss of appetite with reduced drink and food intake also tube feeding or total parenteral nutrition

4 life-threatening

Current analgesic use

0 none

1 topical anesthetic

2 peroral non-steroidal anti-inflammatory drugs

3 peroral opioids

4 intravenous opioids

Dry mouth

0 none

1 symptomatic (dry or thick saliva) without significant dietary alteration

2 symptomatic and significant oral intake alteration (e.g. copious water, other lubricants, diet limited to purees and/or soft, moist foods)

3 symptoms leading to inability to adequately aliment orally; IV fluids, unstimulated tube feedings, or TPN indicated

__________________________________________________________________________

**Supplementary Figure 1.**

Referral from

HSCT center

(n = 95)

Missed recruitment (n = 6)

Language/communication problem (n =18)

Pretransplant dental evaluation

(n = 71)

Declined to participate (n =2)

Severe medical complications/died (n = 1)

Conditioning prior to HSCT

(n = 68)

Severe medical complications/died (n = 4)

Patient did not engraft (n = 1)

Engraftment, neutrophils

> 0.5 x 10^9^

(n = 63)

**Supplementary Table 2.**

Multivariate analysis of variables associated with the development of WHO mucositis grades 2–4 in the neutropenia phase of allogeneic hematopoietic stem cell transplant treatment of child and adolescent patients (n = 68).

| **Variables** | **OR** | **95% CI** | **p-value** |
| --- | --- | --- | --- |
| Age (years) | 1.13 | 1.00, 1.29 | 0.052 |
| Underlying Disease |  |  |  |
| Hematologic, malignant | — | — | Ref |
| Hematologic, non-malignant | 0.22 | 0.06, 0.91 | **0.037** |
| Other | 0.15 | 0.01, 1.74 | 0.193 |
| Transplant donor |  |  |  |
| HLA-matched | — | — | Ref |
| HLA-mismatched | 0.21 | 0.06, 0.71 | **0.013** |

OR: Odds ratio; CI: Confidence interval; Mixed Effect Logistic Model with backward elimination process. Removal threshold was set at p > 0.1. Model designed with Age, Gender, Underlying Disease, Donor for transplantation, Stem cell source, Stem cell dose, Conditioning regimen, and GVHD prophylaxis as fixed effects; HLA: human leukocyte antigen
